# HPLC Method Validation for the Estimation of Lignocaine HCl, Ketoprofen and Hydrocortisone: Greenness Analysis Using AGREE Score

**DOI:** 10.3390/ijms24010440

**Published:** 2022-12-27

**Authors:** Tariq Mehmood, Sana Hanif, Faiza Azhar, Ijaz Ali, Ahmed Alafnan, Talib Hussain, Afrasim Moin, Mubarak A. Alamri, Muhammad Ali Syed

**Affiliations:** 1 Department of Pharmaceutics, Faculty of Pharmacy, The University of Lahore, Lahore 54590, Pakistan; 2 Punjab University College of Pharmacy, University of The Punjab, Faisalabad 38000, Pakistan; 3 Faculty of Pharmaceutical Sciences, GC University Faisalabad, Faisalabad 38000, Pakistan; 4 Department of Pharmacology and Toxicology, College of Pharmacy, University of Hail, Ha’il 81442, Saudi Arabia; 5 Department of Pharmaceutics, College of Pharmacy, University of Ha’il, Ha’il 81442, Saudi Arabia; 6 Department of Pharmaceutical Chemistry, College of Pharmacy, Prince Sattam Bin Abdulaziz University, Alkarj 11323, Saudi Arabia

**Keywords:** HPLC Method validation, ICH guidelines, triple drug, lignocaine HCl, hydrocortisone, ketoprofen, greenness determination

## Abstract

In the current study, the reversed-phased high-pressure liquid chromatography (RP-HPLC) method was proposed for the estimation of lignocaine hydrochloride (LIG), hydrocortisone (HYD) and Ketoprofen (KET) according to International Conference for Harmonization (ICH) Q2 R1 guidelines, in a gel formulation. The chromatographic evaluation was executed using Shimadzu RP-HPLC, equipped with a C8 column and detected using UV at 254 nm wavelength, using acetonitrile and buffer (50:50) as a mobile phase and diluent, at flow rate 1 mL/min and n injection volume of 20 μL. The retention time for LIG, HYD, and KET were 1.54, 2.57, and 5.78 min, correspondingly. The resultant values of analytical recovery demonstrate accuracy and precision of the method and was found specific in identification of the drugs from dosage form and marketed products. The limit of detection (LOD) for LIG, HYD, and KET were calculated to be 0.563, 0.611, and 0.669 ppm, while the limit of quantification (LOQ) was estimated almost at 1.690, 1.833, and 0.223 ppm, respectively. The AGREE software was utilized to evaluate the greenness score of the proposed method, and it was found greener in score (0.76). This study concluded that the proposed method was simple, accurate, precise, robust, economical, reproducible, and suitable for the estimation of drugs in transdermal gels.

## 1. Introduction:

Dermatological disorders such as eczema and dermatitis, caused by pathogens, can be acute by scratching or picking the infected areas of the skin that might be painful for the patient [1]. Various topical dosage forms containing steroids, anesthetics, analgesics in combination with antibiotics are used for the treatment of such diseases [2,3]. It not only reduces the pain but also minimizes the disorder-related symptoms such as edema and itching.

Lignocaine (LIG), which is an amide type anesthetic, is a local anesthetic that numbs the tissues in a target site [4]. It functions by inhibiting the transport of sodium ions via the voltage-gated channels, thereby, it relieves the symptoms of pain as it is locally released from the delivery [5]. Methods have been reported in the literature for the detection of LIG. Numerous instrumentation techniques are reported in literature for the detection of the drug through liquid chromatography equipped with a UV detector and MS/MS spectrometer [6,7], UV spectrophotometry [8], and capillary electrophoresis [9].

Similarly, the Ketoprofen (KET) drug (2-(3-benzoylphenyl)-propionic acid) is one of the effective NSAIDs that has significant importance in joint pain therapy [10,11]. It is advantageous over other NSAIDs because it is non-sedative and lacks addictive potential [12]. It is a white crystalline powder that is freely soluble in organic solvents such as alcohol, dichloromethane, and acetone while insoluble in water [13]. A thorough existing literature survey documented that some techniques that include UV-spectrophotometry [14,15], HPLC [16,17], micellar electrokinetic chromatography [18], capillary zone electrophoresis [19], polarography [20], quantitative Fourier transformation infrared spectrophotometry [21], and electrochemical methods [22] are available for the estimation of KET.

The pharmaceutical products containing corticosteroids in combination with local anesthetics and analgesics have been used in the form of tablets, creams and injections respectively [23]. Hydrocortisone (HYD), a synthetic glucocorticosteroid and a corticosteroid ester play a significant role in the reduction of inflammation by immune suppression. It is also used for the treatment of various skin disorders such as allergies, eczema, dermatitis, and rashes. It is absorbed and reduces inflammation, itching or redness [24]. The chemical name of the hydrocortisone is 11-β, 17-α, 21-trihydroxypregn-4-ene-3, 20-dione with the chemical formula C_23_ H_32_ O_6_ [25]. It is insoluble in water but soluble in organic solvents [26]. Various methods have been reported on the determination of HYD, alone or in combination with other drugs. For instance, with LIG (200 nm), the analyte was developed using acetone, ammonia, and chloroform using thin layer chromatography [23,27,28]. A method was also found for the estimation of KET and LIG [29], however, no method was found to the best of knowledge of the authors for the simultaneous estimation of forth-mentioned drugs with HYD. However, no method has been developed earlier to separate these drugs in a short time without affecting peak shape and resolution. Henceforth, the current study was the continuation of existing literature regarding method development and validation of the Lignocaine hydrochloride, Ketoprofen and hydrocortisone. Initially, the pre-set optimized instrumental settings wet tests for suitability of the method. Afterwards, it was evaluated for validation parameters according to the ICH guidelines [30]. Then the dissolution test of the dosage form was applied to estimate the concentration of drugs. Eventually, greenness estimation of the developed conditions was performed to evaluate the environmental friendliness score of the devised method.

## 2. Results and Discussion

The current study was designed to devise the analysis time of Lignocaine hydrochloride, Ketoprofen and Hydrocortisone since no method was found in the literature for the simultaneous estimation of drugs. Therefore, the optimization of chromatographic conditions was done to study the impact of diluent concentration in the mobile phase and changing pH. The output of system suitability, validation, application, and greenness is as follows.

### 2.1. Development and Optimization of Method

**Figure 1:**
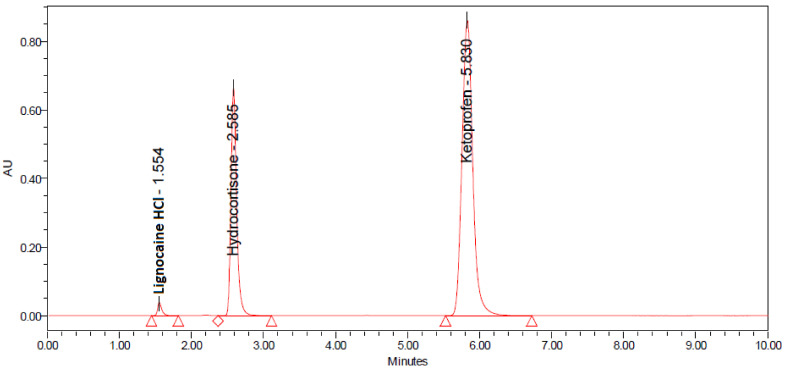
Typical chromatogram depicting the peaks of lignocaine (LIG) HCl, hydrocortisone (HYD) and ketoprofen (KET) for the devised HPLC method at respective concentrations of 80%.

Based on the nature of the analyte, the selection of the HPLC gradient system is the prime and substantial step before the quantitative determination of drug in its commercial dosage. In the current study, reverse phase HPLC method was proposed and optimized using a HPLC system attached with an Agilent^®^ C_8_ column (250 mm × 4.6 mm × 4.6 mm, 5 µm). The chromatogram obtained from the fourth-mentioned instrumental conditions with LIG initially, followed by HYD and KET in the last (Figure 1).

### 2.2. System Suitability

The system suitability was checked by performing the experiment and identifying the changes in separation, asymmetry of the peaks and retention times. The standard solution was injected five times while the sample solution was injected two times. The peak areas, resolution, number of theoretical plates, retention times and peak asymmetry were measured for the standard as well as sample solution. The retention time of LIG, HYD and KET under the optimum conditions were 1.54, 2.57, and 5.78 min respectively. The number of theoretical plates were always over 6000 in all chromatographic runs (Table 1). The peak may be broadened due to multiple factors such as large injection volume, column deterioration, molecular weight of the drug and build-up of contamination in column inlet [31].

The system suitability parameters were tested according to the United States Pharmacopoeia (USP). The working standard solution of Lignocaine HCl, Ketoprofen and hydrocortisone was introduced into HPLC to assess their retention time, and also chromatographic purity and UV spectrum. Once symmetrical peaks were obtained, the five standard injections were injected and their number of theoretical plates, tailing factor, resolution and RSD were calculated. The parameters of system suitability concluded that the chromatographic conditions were satisfactory for the method development and validation. The tailing factor was also found in between 1–2, which is considered acceptable. Similarly, the resolution of the peaks was greater than six. There should be a reasonable value not less than four which could be a possibility that the peaks may be too close to each other. However, if the retention time is too high, for instance greater than 20 min, it may result in a laborious as well as costly analytical procedure. Hence, it is considered good if the peaks are separated from each other that the peaks may be distinctly separated.

### 2.3. Method Validation

The validation factors are as follows.

#### 2.3.1. Linearity

The linearity measured each for LIG and HYD, in the current study, ranged from 0.6 to 56 ppm, and for KET, it was 0.2–100 ppm. A good linear relationship was observed in the analytical calibration curve constructed for Lignocaine hydrochloride, Hydrocortisone and Ketoprofen even though the two latter drugs were hydrophobic and less soluble in pure water. The correlation coefficient was in between 0.9990 to 0.9997, which is considered satisfactory (Table 1).

#### 2.3.2. Accuracy

Accuracy was performed by measuring the analytical concentrations by deliberate addition to the 80% concentration of each of the analyte to form 100% and 120% (Table 2). Outcomes (Table 3) as relative standard deviation have shown a value of less than 2 in all the experimental runs for either analyte. This percentage recovery was within the acceptance criteria of ≤2 [32]. Therefore, the proposed method was found accurate so that it could be further utilized for the estimation of Lignocaine HCl, Hydrocortisone, and Ketoprofen simultaneously.

#### 2.3.3. Precision

Repeatability and intermediate precision were evaluated by five injections of sample solutions at prepared concentrations (Table 2). Response indicated that the RSD values were less than 2% and the proposed method was found to be precise for the quantification of Lignocaine HCl, Hydrocortisone and Ketoprofen (Table 4).

Accuracy and precision were tested at three different concentration levels over a range of 80, 100 and 120%. The obtained percentage recovery was in between 99 to 101%, which was under the acceptance range. Usually, there are three stages of precision i.e., repeatability, reproducibility, and intermediate precision [33]. Repeatability of the proposed method was performed by injecting six sample solutions three times in HPLC. Intermediate precision was carried out on the sample solution on different days by two different analysts. The RSD values were not more than 2% for Lignocaine HCl, Ketoprofen and hydrocortisone which were in accordance with USP i.e., RSD ≤ 2%. It can be observed that the recovery of the analyte was independent of day, analyst, and machine, which concludes that the method was precise.

#### 2.3.4. Robustness

The deliberate changes in chromatographic conditions such as change in pH of the mobile phase (2.5 and 3.5), temperature (20 and 30 °C), and flow rate (0.85 and 1.15 mL/min) were done to detect robustness of the developed method (Table 5). The 100% dose level of the analyte under investigation were selected for calculating robustness. Results has shown that that the recovered analytical concentration of drugs was unaffected by small changes in the conditions and no significant change was noticed in the chromatogram. Henceforth, the developed method was found to be robust with the exception that when the wavelength was changed to above and below the integral whole numbers (nm), issues of detection were formed. Therefore, we can report that the working wavelength (λ) was found to be effective in accurate recovery of the analyte.

#### 2.3.5. Specificity

Specificity of the proposed method was assessed by evaluating the interference of excipients used in the pharmaceutical dosage form as placebo formulation. It was found from the sample run of placebo that when hydroxylpropylmethyl cellulose and Carbopol^®^ containing formulations were analyzed, no interfering peak was observed at the peak retention times of either drug. It added to the findings that the proposed method could be utilized for the estimation of Lignocaine HCl, Hydrocortisone, and Ketoprofen in transdermal delivery (Figure 1).

#### 2.3.6. LOD and LOQ

The LOD and LOQ was measured statistically on the basis of the calibration curve data [34,35]. The measured LOD values for LIG, HYD, and KET were found to be 0.563, 0.611, and 0.669 ppm, whereas the corresponding LOQ values were for 1.690, 1.833, and 0.223 ppm for LIG, HYD, and KET (Table 2).

### 2.4. Greenness Evaluation Using AGREE^®^ Score

**Figure 2:**
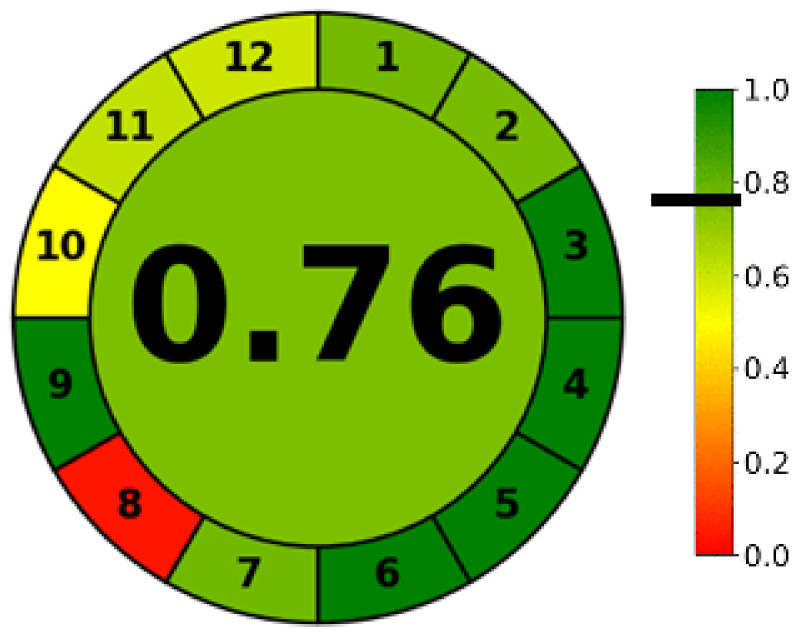
Greenness of the proposed HPLC method using AGREE score.

The greenness is an assessing procedure to demonstrate the extent of greenness present in the sample. Although it was not a mandatory parameter to be tested for the validation, it was performed additionally on the instrumental conditions to ensure that the developed method was not too toxic or dangerous when evaluated on ecological scale. It is a safer prerequisite to estimate the method both for validation as well as for safety extent before using it practically, though the theoretical guidelines have been detailed in the literature [36]. An AGREE pictogram is more meaningful since it uses contour to score an element or parameter and it defines the aggregated safety score after penalties reduction to deviate from safety. Generally, a score above 0.7 is considered green as depicted in the scale (Figure 2). Out of the responses of 12 parameters pertaining to the developed instrumental method, it was found that the AGREE score was in close agreement with the green zone as pointed with the black line in the scale which depicts that the developed method was safer to the environment.

### 2.5. Application to Transdermal Gels

The formulated gels, as prepared by the authors, were evaluated in terms of drug concentrations and it was found that the method was simple and made it easy determine the drugs from the aliquots of dissolution sampling at defined intervals. The drug release data however, will be presented as a separate study by the authors. Moreover, the peak times remained undisturbed when compared with standard values. Moreover, when the drugs in the marketed product were analysed, the analytical concentration of drugs were in accordance with the compendial limits in USP. The amount found for each drug separately in commercial dosage forms contained Lignocaine HCl, Ketoprofen and Hydrocortisone in the respective amounts of 99.80, 100.09 and 97.38% (Table 6).

## 3. Materials and Methods

### 3.1. Materials

Lignocaine (LIG) as hydrochloride (HCl) pure was kindly donated by Hebei Guanlang Biotechnology^®^ Co., Ltd. (Hebei, China), while hydrocortisone (HYD) and ketoprofen (KET) were generously delivered by Selmore^®^ Pharma Pvt. Ltd. (Lahore, Pakistan). The respective drugs were procured from XiaoGan ShenYuan ChemPharm^®^ Co., Ltd. (XiaoGancity, Hubei, China) and Reyoung Pharmaceutical^®^ Co., Ltd. (Shandong, China). Likewise, potassium dihydrogen phosphate and acetonitrile were purchased from Fluka^®^ (Buchs, Switzerland) and ISOLAB^®^ (Eschau, Germany), respectively. All other chemicals, reagents and/or solvents were of analytical HPLC grade and used as received. Similarly, deionized distilled water was used throughout the experiment unless otherwise described.

### 3.2. HPLC Instrumental Conditions

Briefly, Shimadzu^®^ LC20 pump (Machine 1 and 2) as well as Waters^®^ 2695 (Machine 3 and 4) were utilized for optimizing instrumental conditions and HPLC method conditions for the drugs. For analysis, the gradient mobile phase was employed with a rheodyne type sample injector and equipped with an SPD-20 A variable wavelength detector. To accomplish this, the temperature of the Agilent^®^ C_8_ A2010150 X046 (150 mm × 4.6 mm, 5 µm) column was maintained at 30 °C with a flow of the mobile phase at 1 mL/min from the column. The injection volume of sample was preset at 20 µL. The elution was detected at 254 nm with a runtime for 10 min. The data integration and acquisition was performed via Lab solutions^®^ software version 3.2.

### 3.3. Preparation of Mobile Phase and Standard Solution

The solvent mixture as mobile phase comprised of an equal volumetric ratio of acetonitrile and phosphate buffer (pH 3.0) under ambient conditions, where the phosphate buffer was prepared by dissolving 7.2 g of potassium dihydrogen phosphate in 500 mL of deionized water. The final pH was adjusted to 3.0 with 10% *o*-phosphoric acid.

The standard solution was prepared by dissolving 20 mg each for LIG as well as HYD and 50 mg for KET in 100 mL of a volumetric flask containing deionized water (labelled as ‘A’). Then 5 mL of ‘A’ was transferred in a 25 mL of volumetric flask and the remaining volume was filled with the mobile phase. The solution was then filtered through 0.45 µ Millipore^®^ syringe filters before analysis.

### 3.4. System Suitability Parameters

The developed analytical method was then validated in accordance with International Conference of Harmonization (ICH) Q2 R1 guidelines. To be brief, the system suitability was evaluated by identifying the retention time, tailing factor, and resolution of the peaks as well as the number of theoretical plates (USP) [37]. According to United States Pharmacopoeia, the tailing factor (Tf) as well as coefficient of the peak symmetry were calculated (Equation (1)):

(1) $$\mathrm{Tf}=\frac{\left(a+b\right)}{2a}$$

where ‘*a*’ is the distance from the front half of the peak to the peak midpoint (upright from the peak highest point) that is evaluated at 5% of peak altitude and *b* is the distance from the peak center (perpendicular from the peak maximum point) to the trailing edge of the peak estimated at 5% of peak height [38]. Similarly, resolution between the peaks were estimated using Equation (2):

(2) $$\mathrm{Rs}=\frac{\left(\mathrm{tR}2-\mathrm{tR}1\right)}{0.5\left(\mathrm{tW}1+\mathrm{tW}2\right)}$$

where tR2 and tR1 are the retention times of drug 1 and drug 2 for respective peaks 1 and 2. Whereas, tW1 and tW2 are the corresponding peak width.

### 3.5. ICH Validation Parameters

The ICH guidelines provide a detailed procedure for estimating the validation of the devised method. It includes linearity, accuracy, precision (reproducibility and repeatability), limit of detection (LOD), limit of quantification (LOQ), robustness, and specificity [39].

#### 3.5.1. Linearity

Linearity was measured by injecting series of solutions diluted from the standard solution ‘A’ to prepare a desirable analytical concentration and peak area were then determined respectively. The concentration ranges prepared in the study for the linearity range determination has been tabulated in Table 2.

#### 3.5.2. Accuracy

The accuracy of the devised method was measured by estimating the percentage recoveries of the solutions presented in Table 1, which shows the percentage of dose prepared at different levels. Briefly, the amount of drug concentration was deliberately added to the 80% dose solution to form 100% and 120% concentrations. It was then estimated using the devised methods and outcome was analyzed.

#### 3.5.3. Precision

The precision was measured by analyzing the samples at define concentrations of each drug simultaneously i.e., 80%, 100%, and 120% (Table 1). For repeatability and reproducibility, the analysis was performed on different instruments, at different days and by two or more independent analysts [40].

#### 3.5.4. Robustness

To assess the robustness, deliberate variations in the processing conditions were formed, which were minute changes in pH, temperature, flow rate and mobile phase composition.

#### 3.5.5. LOD and LOQ

The limit of quantification (LOQ) and limit of detection (LOD) parameters was measured from the lowest concentration prepared in the study. The LOD (Equation (3)) and LOQ (Equation (4)) were evaluated as follows.

(3) $$\mathrm{LOD}=\frac{\sigma }{s}\times 3.3$$

(4) $$\mathrm{LOQ}=\frac{\sigma }{s}\times 10$$

The ‘s’ is slope of the calibration curve and ‘σ’ is the standard deviation of the lowest concentration of the analyte prepared.

#### 3.5.6. Specificity

The specificity of the proposed method was evaluated using a blank mixture of the analytical solution containing no drug. Any peak was analyzed under devised conditions that could possibly appear in place of the retention time of the peaks [41].

#### 3.5.7. Method Application to Transdermal Gels

After the analytical method was validated, the instrumental conditions were applied to determine the concentration from commercial dosage form. To accomplish this, 2 g of transdermal gel containing the drug equivalent to 20 mg each of LIG and HYD and 50 mg of the KET was placed in the dissolution vessel according to the method reported. Briefly, the gels (containing hydroxypropylmethyl cellulose) were placed in basket and immersed 900 mL of phosphate buffer solution, adjusted to pH 7.4 at 50 rpm. The temperature of whole system was maintained at 37.5 ± 0.5 °C. At defined intervals (0.5, 1, 2, 4 and 8 h), 5 mL of aliquot was diluted with 25 mL of mobile phase to mark up 25 mL. It was then filtered through 0.45 µm filter papers and analyzed quantitatively.

#### 3.5.8. Statistical Analysis

The statistical analysis was carried out using IBM SPSS v.20 software for various validation parameters. Values of mean as well as related standard deviation with percentage of the concentration of analyte were determined.

#### 3.5.9. Greenness Estimation

By using AGREE^®^ software v0.5 beta (Universida de Vigo), the agreement of the recognized analytical procedure for greenness was estimated [41]. Different factors linked with the extent to waste substances, toxic materials and different resources were measured with an already existing standard and penalty on various twelve parameters [36]. After subtracting penalty points, the acquired scores were displayed at the center of a spherical pictogram. Green color referred the safety of method developed to the environment while red color showed the method is not safer to the environment (Figure 2).

## 4. Conclusions

Herein we proposed a RP-HPLC method for the simultaneous quantification of Lignocaine hydrochloride, Ketoprofen, and Hydrocortisone which was not previously found in the literature. The instrumental conditions were evaluated for validation parameters and were found to be accurate, precise, subtle, economical, time saving, and robust. The method provided reasonable resolution and acceptable peak parameters according to USP specifications for lignocaine hydrochloride, ketoprofen, and hydrocortisone. Moreover, the validated method was found environment friendly when compared on a greenness scale as part of ecological safety. The developed method was also tested on self-prepared formulation as it demonstrated no interfering peak of the polymers. Furthermore, the method also detected the analyte in marketed brands which depicts that the validated method can play a significant role in the quantification of triple drugs simultaneously named as Lignocaine hydrochloride, Ketoprofen, and Hydrocortisone. It can be utilized for analysis of dosage forms (bulk or finish) in the pharmaceutical industry and during stability studies with a single run time of <10 min.

## Figures and Tables

**Table 1:** System suitability and limit parameters of the proposed method.

Parameters	LIG	HYD	KET
**System suitability**			
Retention time (min)	1.54	2.57	5.78
Tailing factor	1.39	1.19	1.10
Plate count (USP)	6167	6094	6003
Resolution (USP)	-	9.4	15.7
**Linearity**			
Linear function	y = 3175.2x + 116.67	y = 1 × 10^6^x + 526,211	y = 6 × 10^6^x + 3 × 10^6^
Coefficient of linear regression (r^2^)	0.9994	0.9990	0.9997
Linearity range (ppm)	0.6–56	0.6–56	0.2–100
LOD (ppm)	0.563	0.611	0.223
LOQ (ppm)	1.690	1.833	0.669

**Table 2:** Theoretical contents and dose levels of analyte prepared in the study against the respective mean area of retentive peaks at 100% analytical concentration.

Parameters	LIG		HYD		KET	
Dose Level (%)	Theoretical Contents (ppm)	Mean Area (uA)	Theoretical Contents (ppm)	Mean Area (uA)	Theoretical Contents (ppm)	Mean Area (uA)
80	16	105,831.08	16	3,411,719	40	5,949,786
100	20	132,123.7	20	4,259,328	60	7,446,541
120	24	158,540.0	24	5,111,193	80	8,861,380

**Table 3:** Accuracy of the devised method at 80, 100, and 120% analytical concentrations.

Accuracy	LIG		HYD		KET	
Concentration Level (%)	Amount (ppm)	Recovery % ± RSD	Amount (ppm)	Recovery % ± RSD	Amount (ppm)	Recovery % ± RSD
80	15.91	99.38 ± 0.62	16.0	100.00 ± 1.16	40.25	100.50 ± 1.44
100	20.0	100.0 ± 0.46	20.14	100.50 ± 0.91	60.30	100.20 ± 1.58
120	23.93	99.58 ± 0.51	24.01	100.00 ± 1.29	79.86	99.83 ± 0.38

**Table 4:** Precision of the current HPLC method indicated at different days and analysts.

**Machine/Day 1 & 2**	**Analyst 1 & 2**	**LIG**		**HYD**		**KET**	
**Concentration Level (%)**		**Amount (ppm)**	**Recovery % ± RSD**	**Amount (ppm)**	**Recovery % ± RSD**	**Amount (ppm)**	**Recovery % ± RSD**
80		15.84	99.01 ± 1.28	16.05	100.32 ± 1.76	39.63	99.08 ± 1.08
100		19.87	99.39 ± 1.47	19.16	99.58 ± 0.73	59.68	99.47± 0.97
120		24.18	100.76 ± 1.10	23.80	99.19 ± 0.90	80.08	100.10 ± 0.58
**Inter day precision**							
**Machine/Day 3 & 4**	**Analyst** **3 & 4**	**LIG**		**HYD**		**KET**	
**Concentration level (%)**		**Content recovered** **(ppm)**	**Recovery % ± RSD**	**Content recovered** **(ppm)**	**Recovery % ± RSD**	**Content recovered** **(ppm)**	**Recovery % ± RSD**
80		15.99	99.95 ± 0.92	16.09	100.11± 0.37	39.89	99.74 ± 1.18
100		19.94	99.71 ± 1.46	19.85	99.27 ± 1.77	60.18	100.30 ± 0.75
120		23.87	99.47 ± 0.60	23.96	99.85 ± 0.91	79.34	99.18 ± 0.31

**Table 5:** Outcome of robustness of the proposed method.

Parameters	LIG % ± RSD	HYD % ± RSD	KET % ± RSD
Optimized conditions at 100% analytical dose = pH 3.0, 30 °C and 1.00 mL/min			
**Flow rate**			
0.85 mL/min	100.33 ± 0.95	100.21 ± 1.67	99.15 ± 1.24
1.15 mL/min	98.89 ± 1.36	99.57 ± 1.18	99.90 ± 0.66
**Temperature range**			
20 °C	100.26 ± 1.74	100.49 ± 1.14	100.14 ± 0.86
30 °C	100.58 ± 1.09	99.41 ± 0.68	98.69 ± 0.70
**Mobile phase**			
pH 2.5	99.51 ± 1.38	98.47 ± 1.62	99.35 ± 1.41
pH 3.5	98.86 ± 0.91	100.01 ± 0.75	99.74 ± 0.58

**Table 6:** Assay of commercial tablets according to the developed method.

Active	Brand	Labelled Claim	Amount Found (%)	USP Interpretation
Lignocaine HCl	Lignocaine Gel^®^	2.0% *w*/*v*	99.80 ± 0.83	in limits
Ketoprofen	Profenid Gel^®^	2.5% *w*/*v*	100.09 ± 1.07	in limits
Hydrocortisone	Hydrocortisone 1% Gel	1.0% *w*/*v*	97.38 ± 2.15	in limits

## Data Availability

All data contained in the manuscript.
